# St. Jude Medical Trifecta aortic valve: results from a prospective regional multicentre registry

**DOI:** 10.1186/s13019-015-0379-6

**Published:** 2015-11-20

**Authors:** Giovanni Mariscalco, Silvia Mariani, Samuele Bichi, Andrea Biondi, Andrea Blasio, Paolo Borsani, Fabrizio Corti, Benedetta De Chiara, Riccardo Gherli, Cristian Leva, Claudio Francesco Russo, Giordano Tasca, Paolo Vanelli, Ottavio Alfieri, Carlo Antona, Germano Di Credico, Giampiero Esposito, Amando Gamba, Luigi Martinelli, Lorenzo Menicanti, Giovanni Paolini, Cesare Beghi

**Affiliations:** Department of Cardiovascular sciences, Clinical Science Wing, University of Leicester, Glenfield Hospital, Leicester, United Kingdom; Cardiac Surgery Clinic, University of Milano-Bicocca, San Gerardo Hospital, Monza, Italy; Section of Cardiac Surgery, Department of Cardiovascular Disease, Cliniche Humanitas Gavazzeni, Bergamo, Italy; Department of Cardiac Surgery, IRCCS Policlinico San Donato, Milan, Italy; Department of Cardio-Thoracic and Vascular Surgery, Università Vita-Salute San Raffaele, Milan, Italy; Cardiac Surgery Unit, Cardio-Thoraco-Vascular Department, Niguarda Cà Granda Hospital, Milan, Italy; Department of Cardiac Surgery, Ospedale Civile di Legnano, Legnano, Italy; Cardiac Surgery Unit, Alessandro Manzoni Hospital of Lecco, Lecco, Italy; Cardio-Cerebro-Vascular Department, ‘L. Sacco’ University General Hospital, Milan, Italy

**Keywords:** Aortic valve replacement, Biomaterials, Heart valve bioprosthesis, Heart valve replacement

## Abstract

**Background:**

The Trifecta aortic bioprosthesis (St. Jude Medical, Inc., St. Paul, MN, USA) is a stented pericardial heart valve with excellent preliminary results. Aim of the study was to evaluate its early clinical and hemodynamic performances in a multicenter regional registry.

**Methods:**

Between January 2011 and June 2012, 178 consecutive patients undergoing aortic valve replacement with the Trifecta bioprosthesis were prospectively enrolled at 9 Italian centers. Clinical and echocardiographic data were collectedat discharge, 6-months and at 1-year postoperatively.

**Results:**

The average age was 75.4 ± 7.7 years,and 95 (53 %) were men. Indication for valve replacement included stenosis in 123 patients (69 %), mixed lesions in 25 (14 %), and regurgitation in 30 (17 %). Ninety-three (52 %) patients were in NYHA functional class III/ IV. Hospital mortality accounted for 5 (2.8 %) patients. No valve-related perioperative complications were encountered. Median follow-up was 20.5 months (range: 1-34). Early (≤6 months) complications included one thromboembolic event, one major bleeding, and 3 endocarditis (2 explants). Two late (>6 months) thromboembolic events and two endocarditis (1 explant) were registered. No valve thrombosis or structural deterioration were observed after discharge. At 30-months, freedom from all-cause mortality was 87 %, freedom from valve-related mortality 99.4 %, freedom from endocarditis 97.5 %, and freedom from valve explants 98 %. At 1-year, mean gradients ranged from 8 to 16 mmHg, and effective orifice area indexes from 1.0 to 1.2 cm^2^/m^2^ for valve sizes from 19 to27 mm, respectively. No patients had severe prosthesis-patient mismatch.

**Conclusions:**

Trifecta bioprosthesis provided favourable clinical and hemodynamic results over time.

## Background

The Trifecta aortic bioprosthesis (St. Jude Medical, Inc., St. Paul, MN, USA) is atri-leaflet stented pericardial valve designed for the aortic supra-annular placement [1]. Preliminary and early results about the performances of the Trifecta valve are encouraging [1–5]. Outstanding transvalvular gradients, excellent effective orifice area (EOA) data, low incidence of prosthesis-patient mismatch (PPM), also in patients with a small aortic annulus relative to body size or during exercise and recovery, have been reported [1–13]. On the other hand, given the fact that the Trifecta valve has been introduced in the routine surgery quite recently, large trials and long-term follow up data are still lacking, especially concerning the structural deterioration and the hemodynamic performance of the valve over time [14–16].

Therefore, the present study aims to evaluate early clinical and hemodynamic performance of the Trifecta bioprosthesis in a prospective regional Italian multicenter registry.

## Methods

### Patient selection

Between 1^st^ January 2011 and 30^th^ June 2012, all consecutive patients who received a St. Jude Trifecta valve in aortic position were enrolled at nine Italian centres located in the Lombardia region (Italy). Inclusion criteria considered all the patients undergoing primary aortic valve replacement (AVR) as isolated procedure or in combination with other cardiac surgical procedures. Ethic approval was granted by local Institutional Review Boards, and the informed consent was collected from all participants.

### Surgical technique

Preoperative, anesthetic and postoperative management followed each institutional policy and remained consistent over the study period. Surgery was performed through a median sternotomy or a mini-sternotomy approach with a “J” incision. Cardiopulmonary bypass (CPB) and cannulation techniques were defined according to the required surgical procedure. Mild-to-moderate systemic hypothermia (32°–34 °C) or normothermia were applied. Myocardial protection was achieved according to routine protocols of each institution. Right superior pulmonary vein or main pulmonary artery venting wereused. After the excision of the native aortic valve or the previous aortic valve prosthesis and the decalcification of the aortic annulus, the commercial sizer provided by the manufacturerwas used to choose for correct valve size. Infra-annular or supra-annular implantation techniques as well as interrupted or continuous sutures were performed following surgeon’s preference. Generally, concomitant procedures accounted for coronary artery bypass grafting, proximal aorta surgery, mitral valve repair or replacement.

### Data collection and follow-up

Clinical and echocardiographic data were recorded in a prospective ad hoc database. All surviving patients were postoperatively contacted and the follow-up was performed at the local investigating sites by clinical evaluation and echocardiograms at 6 months and 1 year postoperatively. Where the follow-up was not possible (deceased patients), medical data were collected by telephone interviews of family members and/or confirmed or clarified by general practitioners. Adverse events were classified according to the standardized definitions from the Society of Thoracic Surgeons/American Association for Thoracic Surgery “*Guidelines for reporting morbidity and mortality and cardiac valve interventions*” [17]. Events were classified as occurring early (≤6 months after bioprosthetic implant) or late (>6 months). Follow-up was closed on 30^th^ June 2013.

### Echocardiographic data

Transthoracic echocardiography data were recorded preoperatively, at discharge, as well as 6 months and 1-year postoperatively. Standard prosthetic valve measurements were obtained according to the criteria of the American Society of Echocardiography [18]. Peak and mean transvalvular gradients, EOA, EOA index (EOAI), left ventricular (LV) ejection fraction, end diastolic LV diameter, LV mass, and LV mass index were all recorded. Aortic valve regurgitation was classified as none (0/4), trivial (1/4), moderate (2/4), moderate to severe (3/4) and severe (4/4) according to the width of the regurgitation jet compared to that of the outflow tract [19]. Finally, valve PPM was defined as moderate (EOAI > 0.60 cm^2^/m^2^ and ≤ 0.85 cm^2^/m^2^) and severe (EOAI < 0.60 cm^2^/m^2^) as previously reported [20].

### Statistical analysis

Extracted database variables were tabulated using Microsoft Excel (Microsoft Corp). Statistical analysiswas computed using SPSS, release 22.0 for Windows (IBM SPSS, Inc., Chicago, IL, USA). Continuous datawere expressed as mean ± SD, or median and interquartile range (IQR). Percentages weredetermined for categorical variables. Differences regarding repeated echocardiographic parameters were evaluated with Kruskal-Wallis one-way analysis of variance. Late adverse event rate were determined by Kaplan-Meier method. A *P* value less than 0.05 was considered statistically significant.

## Results

### Population and operative data

The study population included 178 patients, with an average age of age of 75.4 ± 7.7 years (range, 44 to 86 years) and included83 (46.6 %) women. Demographic and preoperative data are listed in Table 1. New York Heart Association (NYHA) class III was present in 78 (43.8 %) of the patients, whereas NYHA class IV in 15 (8.4 %). Mean logistic EuroSCORE was 7.7 ± 6.7 % (IQR, 3 to 11 %). Indications for AVR included stenosis in 123 patients (69.1 %), predominant regurgitation in 30 patients (16.9 %), and mixed disease in 25 patients (14 %). Calcified or degenerative disease of the aortic valve accounted for 155 (87.1 %) cases.

**Table 1:** Preoperative patients details

Variables^a^	All implants (*n* = 178)
*Demographics*	
Age, yrs	75.4 ± 7.7 (72-81)
Female, n (%)	83 (46.6)
BSA, m^2^	1.60 ± 0.33 (1.38-1.79)
*Cardiac presentation*	
Previous AMI, n (%)	22 (12.4)
CAD, n (%)	68 (38.2)
History of AF, n (%)	25 (14.0)
Preoperative NYHA, n (%)	
Class I	13 (7.3)
Class II	72 (40.4)
Class III	78 (43.8)
Class IV	15 (8.4)
*Comorbidities*	
Hypertension, n (%)	136 (76.4)
Diabetes, n (%)	49 (27.5)
COPD, n (%)	24 (13.5)
PVD, n (%)	52 (29.2)
Dyslipidemia, n (%)	83 (46.6)
Renal dysfunction, n (%)	13 (7.3)
Renal failure-dialysis, n (%)	7 (3.9)
Logistic EuroSCORE, %	7.7 ± 6.7 (3.0-11.0)
*Etiology*	
Calcified, n (%)	120 (67.4)
Rheumatic, n (%)	11 (6.2)
Degenerative, n (%)	35 (19.7)
Annular dilatation, n (%)	5 (2.8)
Endocarditis, n (%)	7 (3.9)

^a^For continuous variables, mean ± SD (interquartile range); categorical data, count (percentage)

*AF* atrial fibrillation, *AMI* acute myocardial infarction, *BSA* body surface area, *CAD* coronary artery disease, *COPD* chronic obstructive pulmonary disease, *NYHA* New York Heart Association, *PVD* peripheral vascular disease

Concomitant procedures were performed in 97 (54.5 %) patients, and included concomitant coronary artery bypass graft (CABG) in 63 (35.4 %) cases (Table 2). A mini-sternotomy approach was employed in 10 (5.6 %) patients. The mean CPB and aortic cross clamp time (ACC) time for isolated AVR were 79.9 ± 31.8 and 58.7 ± 25.8 min, respectively. The prosthesis sizes were 19 mm in 31 patients (17.4 %), 21 mm in 58 (32.6 %), 23 mm in 58 (32.6 %), 25 mm in 24 (13.5 %), and 27 mm in 7 (3.9 %).

**Table 2:** Perioperative patients details

Variables^a^	All implants (*n* = 178)
*Valve size implanted,* n (%)	
19 mm	31 (17.4)
21 mm	58 (32.6)
23 mm	58 (32.6)
25 mm	24 (13.5)
27 mm	7 (3.9)
*Operative data*	
Isolated AVR, n (%)	81 (45.5)
CABG, n (%)	63 (35.4)
ACC time, min	67.9 ± 41.4 (45-95)
CPB time, min	103.9 ± 45.7 (70-131)
*Postoperative data*	
IABP, n (%)	6 (3.4)
Re-exploration for bleeding, n (%)	7 (3.9)
Stroke, n (%)	2 (1.1)
Respiratory failure, n (%)	14 (7.9)
Acute kidney injury, n (%)	24 (13.5)
Renal replacement therapy, n (%)	11 (6.2)
Atrial fibrillation, n (%)	79 (44.4)
Ventilation, hours	33.8 ± 98.8 (7-24)
ICU stay, hours	68.5 ± 119.2 (24-60)
Hospital mortality, n (%)	5 (2.8)

^a^For continuous variables, mean ± SD (interquartile range); categorical data, count (percentage)

*ACC* aortic cross clamp time, *AVR* aortic valve replacement, *CABG* coronary artery bypass grafting, *CPB* cardiopulmonary bypass, *IABP* intra-aortic balloon pump, *ICU* intensive care unit

### Early and medium-term clinical outcomes

Hospital mortality accounted for 5 (2.8 %) patients because of low cardiac output syndrome followed by multiorgan failure (*n* = 1) or sepsis (*n* = 4). Re-exploration for bleeding was required in 7 (3.9 %) cases, and 2 (1.1 %) patients had stroke. Hospital stay was 11.1 ± 10.6 days (IQR, 6 to 11 days). No valve-related perioperative complications were recorded. Postoperative complications are depicted in Table 2.

**Fig. 1:**
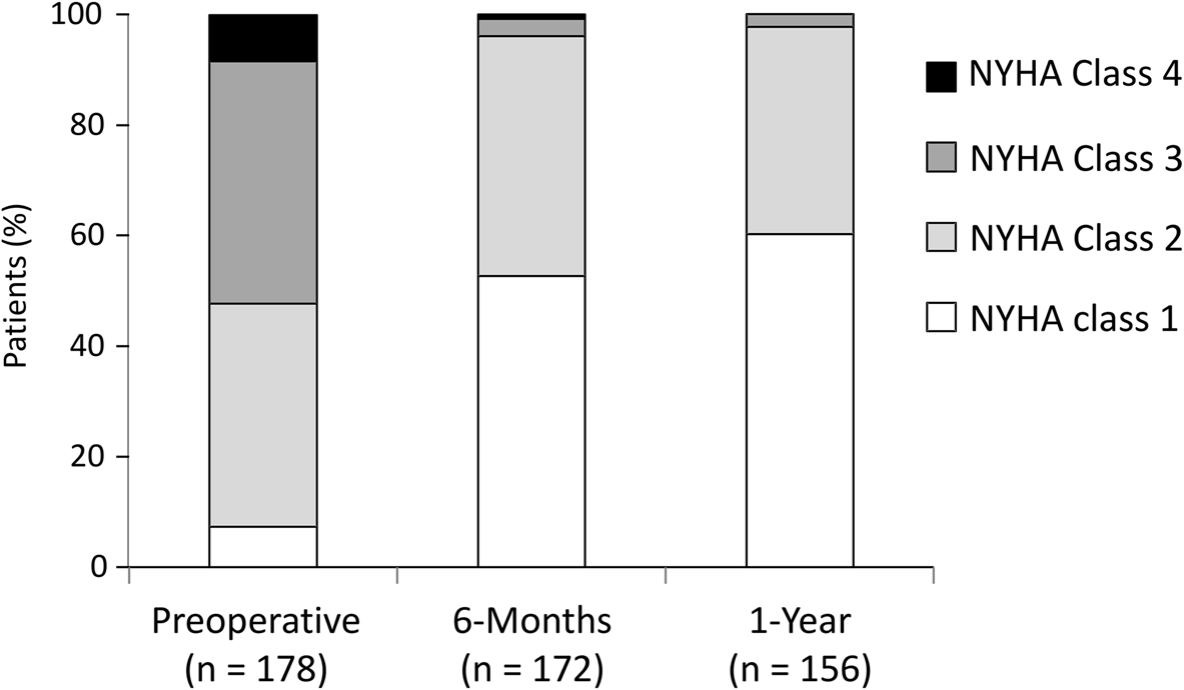
NYHA class. NYHA functional class over time (*P* < 0.001)

Median follow-up was 20.5 months (range: 1-30 months). During the follow-up, patients’ clinical status significantly improved in all cases (*P* < 0.001), and 97.7 % of patients were in classes I or II at 1 year follow-up (Fig. 1).

**Fig. 2:**
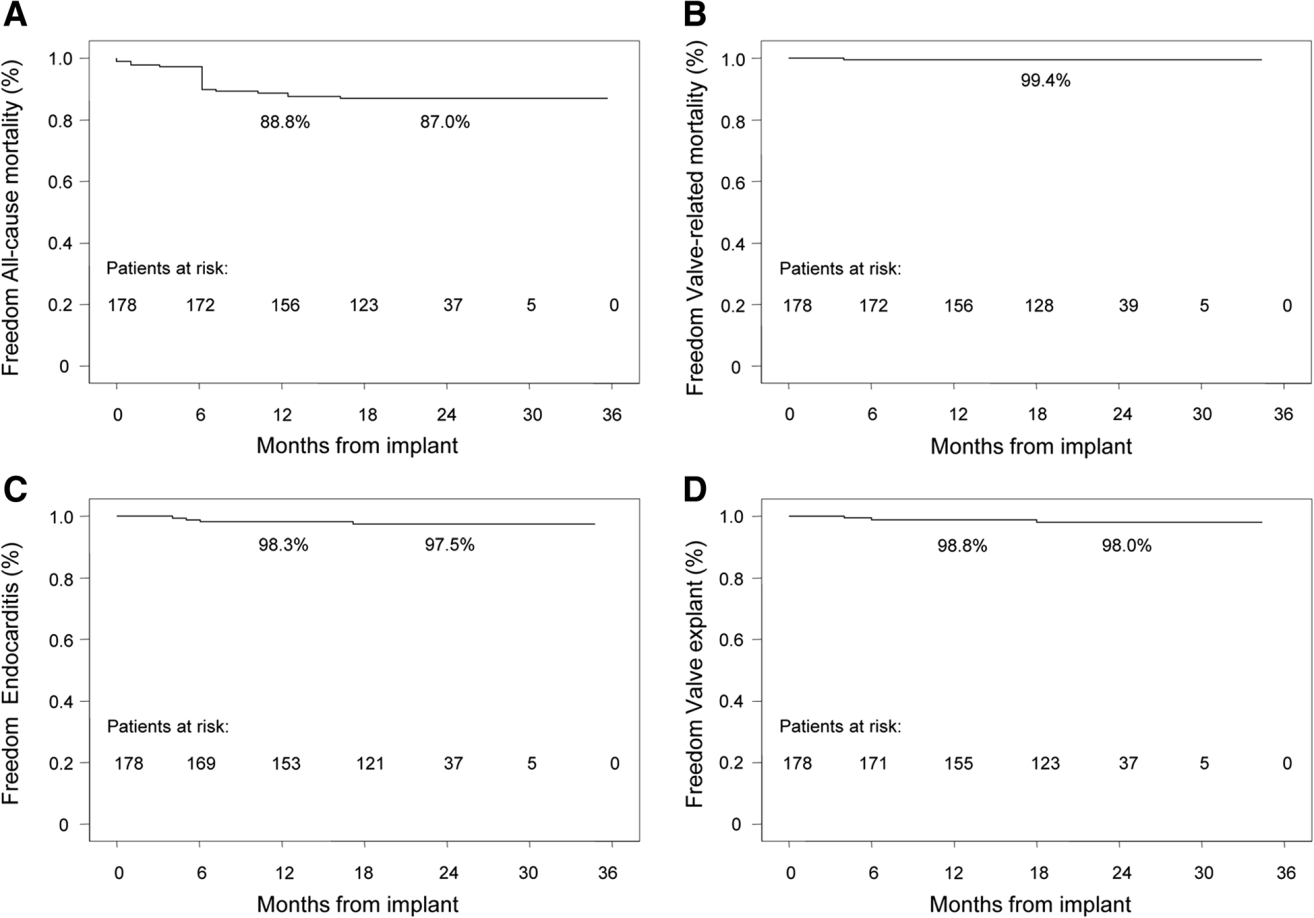
Follow-up data. Freedom from all-cause mortality (**a**), freedom form valve-related mortality (**b**), freedom from endocarditis (**c**), and freedom from valve explant (**d**)

Early (≤6 months) and late (>6 months) adverse events were all recorded (Table 3). There was only one early thromboembolic event which caused a transient ischemic attack and two late thromboembolic events which led to stroke. Three endocarditis occurred within six months from the operation and two of them required prosthesis explant. Two further endocarditis (1 explant) were recorded during the late follow-up. None of the registered endocarditis were primarily operated on for native valve endocarditis. No valve thrombosis, hemolysis and structural valve deterioration were registered after discharge. Overall, freedom from all-cause mortality was 87.0 ± 2.5 % at 30 months, whereas freedom from valve related mortality was 99.4 ± 0.6 %, freedom from endocarditis was 97.5 ± 1.2 %, and freedom from valve explants was 98.0 ± 1.1 % (Fig. 2).

**Table 3:** Early and late adverse events

Variables^a^	Early (≤6 months)	Late (>6 months)
Thromboembolism	1 (0.6)	2 (1.1)
Stroke	0	2 (1.1)
TIA	1 (0.6)	0
Valve thrombosis	0	0
Hemolysis	0	0
Major bleeding	1 (0.6)	0
Non-structural valve dysfunction	0	1 (0.6)
Paravalvular leak		
Minor	6 (3.4)	6 (3.4)
Major	1 (0.6)	0
Structural valve deterioration	0	0
Endocarditis	3 (1.7)	2 (1.1)
Prosthesis explant	2 (1.1)	1 (0.6)
Mortality Valve-related	1 (0.6)	0

^a^Count (Percentage)

### Hemodynamic results

**Fig. 3:**
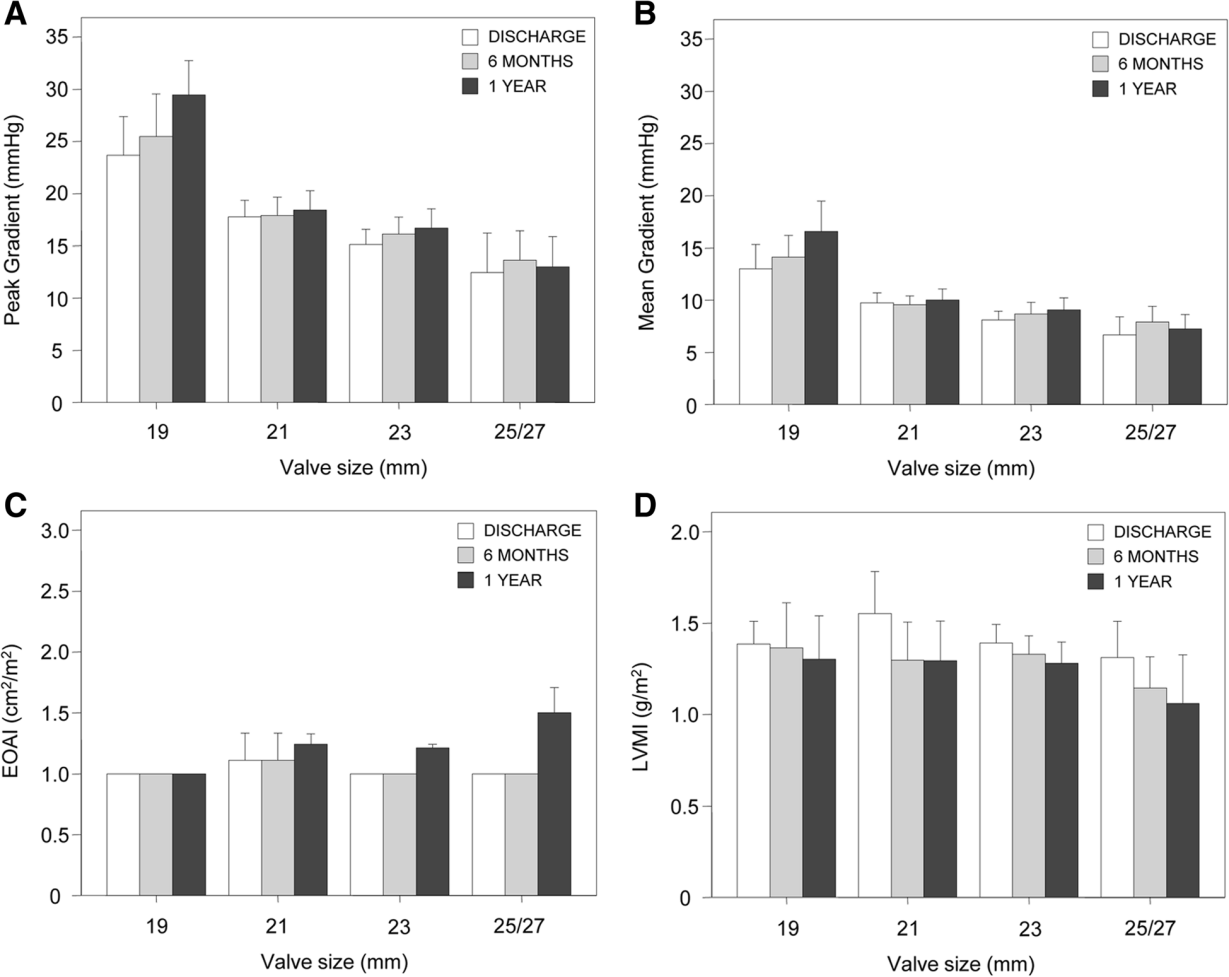
Echocardiographic data. Average peak gradient (**a**), average mean gradient (**b**), average EOAI (**c**), and average LVMI (**d**) over time. EOAI = effect orifice area index; LVMI = left ventricular mass index

Hemodynamic results at discharge, 6 months and 1 year are reported in Table 4 and Fig. 3. Mean and peak transvalvular gradients significantly decreased after AVR, with a significant reduction to approximately more than 50 % of the preoperative values at six months. At discharge, average mean gradients ranged from 7.4 to 13.5 mmHg and average peak gradients ranged from 13.6 to 25.3 mmHg for valve sizes 19 to 25/27 mm, respectively. At 1 year of follow-up, average mean gradients ranged from 7.7 to 16.6 mmHg and average peak gradients ranged from 14.1 to 29.5 mmHg for valve sizes 19 to 25/27 mm, respectively. Significant increases in EOA and EAOI were also observed. At discharge, EOAI ranged from 1.6 to 2.2 cm^2^/m^2^ and from 1.4 to 2.5 cm^2^/m^2^at 1 year for valve sizes 19 to 25/27 mm, respectively. A significant reduction of left ventricular mass index (LVMI - g/m^2^) was documented at discharge (134.3 ± 42.2 g/m^2^), at 6 months (120.9 ± 40.9 g/m^2^), and 1-year (123.2 ± 44.8 g/m^2^). At 6 months, valve prosthesis-patient mismatch was mild-to-moderate in 19 (10.6 %) patients, and severe PPM was not documented in any patient.

**Table 4:** Echocardiographic data over time

Variables^a^	Preoperative	Discharge	6 months	1 year
Size 19 mm (n)	31	29	29	25
Peak gr, (mmHg)^b^	76.1 ± 28.7	25.3 ± 9.5	24.1 ± 8.2	29.5 ± 11.0
Mean gr (mmHg)^b^	45.7 ± 18.3	13.5 ± 5.7	13.2 ± 4.4	16.6 ± 5.8
EOA (cm2)^b^	1.0 ± 0.5	1.6 ± 0.4	1.4 ± 0.4	1.4 ± 0.1
EOAI (cm2/m2)^b^	0.2 ± 0.4	1.1 ± 0.7	1.0 ± 0.1	1.0 ± 0.1
LVMI (g/m2)^b^	136.2 ± 8.2	124.2 ± 23.3	118.1 ± 44.6	123.4 ± 40.4
EF (%)	58.7 ± 9.4	55.5 ± 8.2	57.3 ± 5.9	58.4 ± 6.1
Size 21 mm (n)	58	57	56	51
Peak gr (mmHg)^b^	87.7 ± 25.6	18.2 ± 5.2	17.9 ± 5.2	18.3 ± 5.2
Mean gr (mmHg)^b^	54.7 ± 17.3	9.8 ± 2.9	9.4 ± 2.7	9.9 ± 3.0
EOA (cm2)^b^	0.7 ± 0.2	1.9 ± 0.9	2.0 ± 0.3	2.1 ± 0.5
EOAI (cm2/m2)^b^	0.5 ± 0.1	1.1 ± 0.3	1.2 ± 0.3	1.4 ± 0.4
LVMI (g/m2)^b^	146.1 ± 22.8	142.2 ± 52.3	120.5 ± 48.7	127.7 ± 57.1
EF (%)	58.4 ± 7.9	56.4 ± 8.4	56.8 ± 8.2	55.9 ± 8.4
Size 23 mm (n)	58	56	56	53
Peak gr (mmHg)^b^	77.6 ± 29.9	14.4 ± 5.0	15.1 ± 4.8	16.6 ± 5.4
Mean gr (mmHg)^b^	47.8 ± 20.3	7.6 ± 2.3	8.2 ± 3.1	9.1 ± 3.2
EOA (cm2)^b^	1.1 ± 0.6	2.1 ± 0.3	1.9 ± 0.3	1.8 ± 0.2
EOAI (cm2/m2)^b^	0.6 ± 0.8	1.1 ± 0.3	1.0 ± 0.1	1.2 ± 0.1
LVMI (g/m2)^b^	140.8 ± 25.8	135.2 ± 37.3	123.4 ± 33.2	125.9 ± 30.8
EF (%)	55.7 ± 8.9	52.4 ± 8.9	54.5 ± 9.5	53.4 ± 8.6
Size 25/27 mm (n)	31	31	31	27
Peak gr (mmHg)^b^	62.7 ± 28.9	13.6 ± 5.2	14.4 ± 4.6	14.1 ± 4.7
Mean gr (mmHg)^b^	36.7 ± 18.0	7.4 ± 2.8	8.2 ± 2.5	7.7 ± 2.4
EOA (cm2)^b^	1.2 ± 0.5	2.2 ± 0.8	2.0 ± 0.8	2.5 ± 1.3
EOAI (cm2/m2)^b^	0.9 ± 0.4	1.2 ± 0.4	1.2 ± 0.5	1.5 ± 0.5
LVMI (g/m2)^b^	157.0 ± 53.1	126.2 ± 42.9	118.4 ± 24.3	100.9 ± 41.0
EF (%)	54.5 ± 11.1	50.8 ± 9.7	55.5 ± 8.9	56.2 ± 9.5

^a^Data are expressed as mean ± SD

^b^ *p* < 0.001 between discharge or 6-month or 1-year values versus preoperative values

*EF* ejection fraction, *EOA* effective orifice area, *EOAI* effective orifice are index, *LVMI* left ventricular mass index

Finally, at discharge mild central aortic regurgitation insufficiency was present in 31 (17.4 %) of the patients, while moderate in 3 (1.7 %). No severe central regurgitation was documented over-time. Paravalvular-leak detection is reported in Table 3.

## Discussion

The present regional prospective multicenter registry allowed the authors to record and analyze the early clinical and echocardiographic resultsof the Trifecta bioprosthesisimplanted over a period of 24 months at nine Italian hospital. The follow-up and the data showed a good safety profile of the valve with no valve-related perioperative complications, good perioperative mortality and overall survival, no valve thrombosis, no clinically significant hemolysis or structural deterioration. In addition, the echocardiographic assessment of the hemodynamicperformances of the Trifecta valve revealed nearly physiological data and excellentperformances also when compared with other pericardial prosthesis [6–13].

The increasing need for biological prosthesis related to the rising age of the patients undergoing aortic valve replacement, is pushing biotechnologies toward the research of the ideal prosthetic valve. Such a valve should allow the surgeon to use an easy, quick and safe implant technique with low risk for dehiscence or structural degeneration in the long period [21]. The ideal bioprosthesis should also have a low intrinsic thrombogenicity with no need for the anticoagulation therapy, and a high-quality hemodynamic performance with low gradients, large EOA and good movement and coaptation of the leaflet [1, 21, 22]. The Trifecta bioprosthesis is trying to address those requests with its features, and it has been designed with a concave, scalloped sewing ring for a supra-annular implant with non-everting sutures [1]. Ugur et al. [21] demonstrated that AVR with Trifecta bioprosthesis can be safely performed with non everting pledget-reinforced sutures, everting mattress sutures with or without pledgets, simple sutures or continuous suture techniques giving the surgeon a wide range of choice in terms of implant technique. In our experience, infra-annular or supra-annular implantation techniques as well as interrupted or continuous sutures were performed following surgeon’s preference and the low rate of paravalvular leak confirmed the ease of implantability of the valve. However, an appropriate sizing and annular decalcification are mandatory to avoid paravalvular leaks, as far as the prevention of stent distortion which can abolish the benefits of a cuff designed to conform to the native annulus after implantation [2].

In addition, the nearly physiological hemodynamic performances of Trifecta bioprosthesis could decrease the need of stentless valves which, on the contrary, require a substantial learning curve, technically demanding implantation and an aortic root replacement in case of failure of the prosthesis [23]. Its excellent hemodynamic performances could also simplify the implant process avoiding additional root and annular enlargement [1–5, 24]. As a matter of fact, the external mounting of leaflets allows for a wider opening, and the expansible stentcould limit impedance to flow during high flow conditions as during exercise [12]. The nearly cylindrical opening of the Trifecta bioprosthesis during systole provides gradients and EOAs that result superior to any other available stented aortic prosthesis and approach those of stentless valves [1]. Bavaria et al. [1] provided excellent hemodynamic performances of the Trifecta bioprosthesis in more than 1000 patients enrolled at 31 centers, documenting at the time of discharge an average mean gradients ranging from 9.3 to 4.1 mmHg and an EOA ranging from 1.58 to 2.50 cm^2^ for valve sizes 19 to 29 mm, respectively. Clinically, they also demonstrated a freedom from NYHA class III or IV symptoms of 96.1 % [1]. The present multicenter regional registry confirmed these excellent clinical and hemodynamic performances. In consonance with Bavaria’s study [1], the present one reports data after more than 1-year of follow-up, whereas the majority of the published papers documented the performances of the Trifecta bioprosthesis at discharge or at a maximum follow-up of 6 months [12, 24].

In our multicenter registry, the favorable hemodynamic led to a low incidence of PPM which was mild-to moderate in 19 (11 %) patients only, while severe mismatch was never detected. Literature suggests thatPPM is related to a significant increase in all-cause andcardiac-related mortality over long-term follow-up after AVR,since the persistent LV afterload imposedby PPM may impair the postoperative recovery of the coronaryflow reserve and hinder the regression of LV hypertrophy anddysfunction [25]. In our experience, LVMI significantly decrease from preoperative values up to 1 year after surgery, suggesting a positive effect of the bioprosthetic valve on the myocardial hypertrophy.

Finally, we were able also to confirm the postoperative satisfactory results in terms of valve thrombosis, structural deterioration freedom from all-cause mortality, valve-related mortality, endocarditis and valve explants. Therefore, the present data are comparable to those previously described for the Trifecta bioprosthesis and other bioprosthetic aortic valves [1–13, 26–28]. During the follow-up, we observed five endocarditis with three explant, but no signs of structural valve deterioration were detected. Bavaria et al. [1] reported one explant for early deteriorationover 1014 enrolled patientsandthe explanted valve did not demonstrate thickeningor calcification of the cusps. However, despite the use of anticalcification agents and the elimination of a tacking suture at the top of the commissures so to decrease the risk of tearing, early degeneration is still possible from an accelerated immunologicreaction to the pericardial tissueor because of a reaction to the suture material [14–16].

The present study has limitations. First, it enrolled a relatively small sample size, although it represents one of the largest patient group treated with the Trifecta bioprosthesis to date [1–3]. Second, there is the lack of a control group for comparison with other bioprosthetic and stentless valves, a limitation shared with other studies [1–5]. Third, our patient population is heterogeneous with reference to the surgical access (full-sternotomy *vs* mini-sternotomy) and implant techniques (infra-annular *vs* supra-annular or interrupted *vs* continuous sutures), which constituted a minor possible confounder in clinical and performance assessment of the present bioprosthesis. Finally, the follow-up time is another limitation of the present study since the Trifecta bioprosthesis has been commercialized few years ago, and long-term follow-up data are certainly mandatory to confirm its promising clinical and hemodynamic results.

## Conclusion

In conclusion, the present prospective multicenter regional study provided favourable clinical and hemodynamic results for the Trifecta bioprosthesis, showing ease of implantation, low incidence of early valve degeneration and valve-related morbidity. The Trifecta aortic valve should be considered as a good option and alternative to other biologicalstented aortic valves. However, further studies are mandatory to assess the long-term results, confirming the early documented favorable data.

## Abbreviations

ACC: Aortic cross clamp time
AVR: Aortic valve replacement
CABG: Coronary artery bypass graft
CPB: Cardiopulmonary bypass
EOA: Effective orifice area
EOAI: Effective orifice area index
LV: Left ventricle
LVMI: Left ventricular mass index
NYHA: New York Heart Association
PPM: Prosthesis-patient mismatch
